# Abnormal FHIT expression profiles in cervical intraepithelial neoplastic (CIN) lesions

**DOI:** 10.1038/sj.bjc.6600077

**Published:** 2002-02-01

**Authors:** G Terry, L Ho, P Londesborough, J Cuzick

**Affiliations:** Department of Molecular Pathology, Windeyer Building, University College London, 46 Cleveland Street, London W1T 4JF, UK; Department of Mathematics, Statistics & Epidemiology, Imperial Cancer Research Fund, Lincoln's Inn Fields, London WC2A, 3PX, UK

**Keywords:** CIN, HPV, FHIT

## Abstract

Abnormal fragile histidine triad transcripts were found in 20–30% of CIN2/3 lesions and 11% of normal cervical biopsies by RT–PCR. Bi-allelic loss of the fragile histidine triad gene and the loss of fragile histidine triad protein expression detectable by immunochemical staining with a polyclonal fragile histidine triad specific antibody was rare. The genomic changes showed no association with the presence of human papillomavirus types which carry high risk for cervical cancer (high risk human papillomavirus) as assessed by a type-specific multiplex PCR. The presence of abnormal fragile histidine triad transcripts in a subset of CIN2/3 lesions with no high risk human papillomavirus suggests that this could be an independent risk factor associated with an alternative carcinogenic pathway.

*British Journal of Cancer* (2002) **86**, 376–381. DOI: 10.1038/sj/bjc/6600077 www.bjcancer.com

© 2002 The Cancer Research Campaign

## 

Cervical cancer is the second most common malignancy in women worldwide. It is generally accepted that the development of cervical cancer from precancers (CIN2/3) involves multistep molecular changes and is therefore a preventable disease if precancers are detected and treated early.

The major risk factor for CIN2/3 and cancer is infection with high risk human papillomavirus (HR HPV) types which include HPV16, HPV18, HPV31, HPV33, HPV35, HPV45, HPV51, HPV52, HPV56, HPV58, HPV59 and HPV68. One or more of these virus types have been reported to be present in 90–95% of CIN2/3 lesions and in nearly all cancers (Lorincz *et al*, 1992; Bosch *et al*, 1995; Walboomers *et al*, 1996). However, infection with HR HPV does not invariably lead to the development of high grade cervical lesions and there is a clear requirement for other co-factors, presumably to advance the oncogenic process initiated by HR HPV (zur Hausen, 1999). The effects of co-factors are difficult to measure objectively because of the dominant effects of HR HPV infection but tobacco smoking has consistently been associated with cervical cancer (Szarewski *et al*, 1996) and has been found recently to induce genomic instability in head and neck cancers (Schantz *et al*, 2000). Other potential co-factors include oral contraceptive use and other sexually transmitted diseases.

Genomic instability has been found in cervical neoplasia and commonly involves the short arm of chromosome 3. Results reported so far are based on small numbers of samples and show no clear correlation between genomic changes and lesion grades (Butler *et al*, 2000; Yoshino *et al*, 2000). Allelic loss and microsatellite instability were found to occur in lesions with or without malignant potential in similar proportion (Wistuba *et al*, 1997; Chu *et al*, 1998; Yoshino *et al*, 1998; Butler *et al*, 2000; Lin *et al*, 2000). Inconsistent results were also obtained in the analysis of abnormal transcripts encoded by the fragile histidine triad (FHIT) gene at chromosome 3p14.2. These are also indicative of genomic instability (Sozzi *et al*, 1996) but the incidence reported varies from 30 to 80% in cervical cancer and 0 to 40% in normal tissue (Greenspan *et al*, 1997; Chu *et al*, 1998; Muller *et al*, 1998; Su *et al*, 1998; Yoshino *et al*, 1998; Nakagawa *et al*, 1999; Segawa *et al*, 1999). In only one study, which involved FHIT protein staining in 110 paraffin sections, was a reduction in FHIT gene expression observed to correlate with increasing lesion grade (Birrer *et al*, 1999). How genetic alterations in chromosome 3p interact with HR HPV is also not clear (Muller *et al*, 1998; Butler *et al*, 2000). Integration of HPV16 sequences at the FHIT locus has been described but this does not appear to be site specific (Wagatsuma *et al*, 1990; Wilke *et al*, 1995). HPV16 sequences were not present in abnormal FHIT transcripts (Muller *et al*, 1998) and the presence of HR HPV E6/E7 transcripts and abnormal FHIT transcripts were negatively correlated in cervical cancer (Segawa *et al*, 1999).

In this investigation, abnormal FHIT gene expression based on the detection of abnormal FHIT transcripts or a reduction of immunohistochemical staining for FHIT protein was studied in 281 and 114 biopsies respectively, of normal cervix and CIN lesions. We assessed whether these indicators of genomic instability at chromosome location 3p14.2 were present at an early stage, when the oncogenic potential of HR HPV is not yet fully expressed. The influence of HR HPV on genomic instability was also investigated. The potential implications of abnormal FHIT expression in the pathogenesis of CIN lesions are discussed.

## MATERIALS AND METHODS

### Specimens

All specimens were collected in accordance with the guidelines approved by the Whittington Hospital Trust UK ethics committee.

### HPV and abnormal FHIT transcript status

Nucleic acid preparations from 281 biopsies of normal cervix and CIN lesions from a total of 91 women participating in a previous study of HPV E6 and E7 transcripts (Ho *et al*, 1994) were used. Five cancer biopsies from the same study acted as high grade lesion controls. Routine histology was reviewed by a consultant histopathologist. Nucleic acid extraction was carried out using guanidinium isothiocyanate at 60°C for 10 min to selectively lyse the epithelium and remove it from the underlying, more resistant, stromal tissue. After further extraction with hot phenol/chloroform and digestion with proteinase K (PK, 200 μg ml^−1^ (Sambrook *et al*, 1989)) total nucleic acids were precipitated and stored in 1 ml of ethanol containing 0.3 M sodium acetate (pH 5.2) at −70°C. HPV assays were carried out using 50 μl aliquots which were centrifuged at 14 000 **g** and 4°C for 15 min. The pellet was re-dissolved in 20 μl of TE (10 mM Tris, 1 mM EDTA, pH 8.0). Thirteen HR HPV types were assayed by a multiplex type specific PCR (mts–PCR) using primers that are completely homologous to the corresponding HPV genome sequence (Terry *et al*, 2001). The 3′ end of each primer was not homologous to any other region of any of the 13 genomes. The primers amplified DNA sequences in the E6 or E6/E7 genes, the region most likely to be retained after viral DNA integration.

FHIT transcripts were detected by semi-nested RT–PCR. Nucleic acids from a 500 μl aliquot in ethanol was pelleted, redissolved in TE, digested with RQ1 RNase free DNase I (Amersham Pharmacia Biotech) at 250 μg ml^−1^. The RNA was then phenol-chloroform extracted and re-precipitated with ethanol. FHIT cDNA was synthesised using a Reverse Transcription System kit (Promega) and subsequently amplified for 30 cycles (94°C for 30 s, 60°C for 1 min, 68°C for 2 min) with primers FH203S (forward) and FH1038 (reverse). The efficiency of reverse transcription was monitored using aldolase PCR primers (Chelly *et al*, 1988). The PCR product was diluted 1/10 and amplified in a semi-nested PCR for a further 30 cycles (cycling conditions as above) using primers FH203S (forward) and FH904S (reverse). The FHIT primers were as described by Fong *et al* (1997) but with the addition of 5′-leader sequences (nucleotides in lower case) to allow direct sequencing of the PCR products using available Cy5 labelled sequencing primers for rubella virus (in FH203S) or M13 (in FH904S).


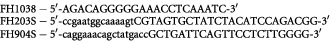


Nucleic acid sequencing was carried out using an ALF-Express automatic sequencer.

### HPV and FHIT protein status

Paraffin blocks from corresponding cases used for the FHIT transcript study were not available due to re-organizations in the National Health Service. For this analysis 114 biopsies of normal cervix and CIN lesions from a total of 42 women participating in two previous studies of cervical cancer risk factors (Mansell *et al*, 1994; Cuzick *et al*, 1996) were used. Nine cancer lesions (Cuzick *et al*, 1996) were included as high grade lesion controls. Routine histology was reviewed by a consultant histopathologist. Sections (3 μm thick) were mounted on coated slides (BDH). For HPV genotyping, individual sections were de-waxed with EZ-DeWax (InnoGenex) according to the instruction of the manufacturer, scraped from the slide, washed three times with ethanol, digested with PK and tested by mts–PCR as described above. For histological and immunohistochemical staining, sections were de-waxed with xylene and stained with either haematoxylin and eosin or FHIT antibody (Zymed). FHIT antibody was used at a dilution of 1/200 after epitope retrieval in accordance with the manufacturer's instructions and its presence detected by reaction with monoclonal alkaline phosphatase conjugated anti-rabbit IgG antibody (gamma chain specific) at a dilution of 1/100 (Sigma A2556) and 5-bromo-4-chloro-3-indolyl phosphate/nitroblue tetrazolium (Sigma B5655) as substrate. Nuclei were stained with 0.1% nuclear fast red in 5% aluminium sulphate. Specific cytoplasmic immunostaining for FHIT protein in cervical lesions was assessed by comparison with normal epithelium (positive control, present in most sections) and with stromal cells (negative control, present in all sections).

## RESULTS

The histological grades and the HR HPV status of the lesions used in this study, subdivided into those used for FHIT transcript or protein detection, are shown in Table 1

**Table 1:**
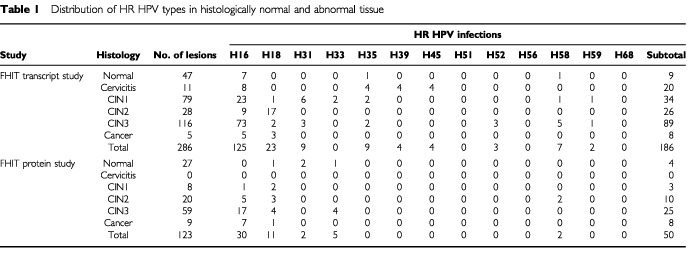
Distribution of HR HPV types in histologically normal and abnormal tissue

. High grade lesions were predominantly associated with HPV16, HPV18, HPV31, HPV33, HPV35 and HPV58 but 20% (29 out of144) were negative for HR HPV. Seven lesions contained more than one HR HPV type. Cancer biopsies were included as positive controls for fully invasive disease and one of these was HR HPV negative. The association of high grade lesions with HR HPV was clearly evident (*P*_trend_<0.001 in the transcript study and *P*_trend_=0.012 in the protein study).

**Figure 1:**
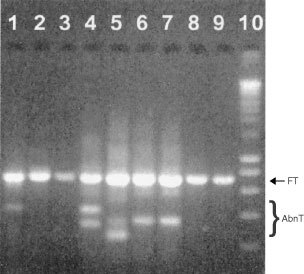
Agarose gel electrophoresis of semi-nested RT–PCR products (lanes 1–9). Size markers in lane 10 are 200, 400, 600, 800, 1000, 1500, 2000, 2500, 3000, 4000, 5000, 6000, 8000 and 10 000 bp from bottom upwards. FT=normally spliced transcript (702 bp). AbnT=abnormal transcripts.

Representative semi-nested FHIT transcript PCR products are shown in Figure 1

**Figure 2:**
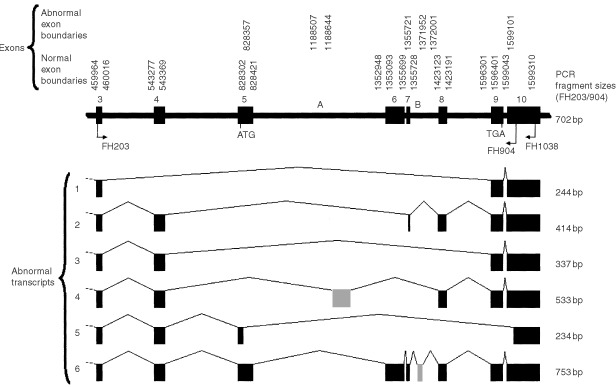
Abnormal FHIT transcripts found in this study and their generation by alternative splice site usage. Only the region amplified by the FH203S/FH1038 primers is shown. The FHIT coding sequence start and stop codons are indicated and the origins of two abnormal exons found (grey) are labelled A and B. Introns are shown on a 500 times smaller scale than exons. Numbering of the first and last bases of exons is from Genbank accession number NT_005607.3.

. Full length FHIT transcripts were not detectable in nine lesions (two cancers, five CIN3 and two CIN1). Abnormal splicing patterns similar to those previously reported (Sozzi *et al*, 1996) were found by nucleic acid sequencing to be responsible for size-shifted PCR fragments. These included (i) skipping of exons 4–8, exons 5–8, exons 5–6 with an alternative splice site within exon 7 or exons 6–9 with alternative splice sites within exons 5 and 10, (ii) replacement of exons 5–7 with 138 bases from intron 5 and (iii) an insertion of a 50 base sequence from intron 7 between exons 7 and 8 (Figure 2

). After adjusting for HR HPV, abnormal FHIT transcript expression (aberrand and absent) was found to be associated with increasing CIN grades (*P*_trend_<0.001) (Table 2A

**Table 2:**
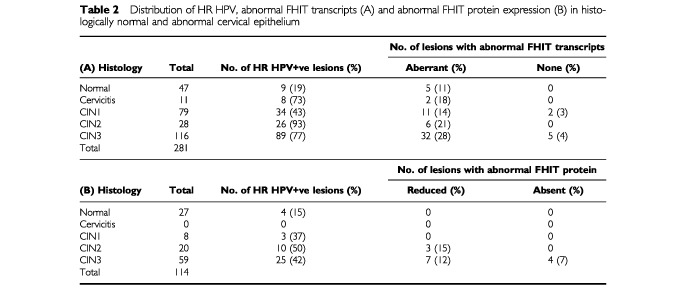
Distribution of HR HPV, abnormal FHIT transcripts (A) and abnormal FHIT protein expression (B) in histologically normal and abnormal cervical epithelium

).

Figure 3

**Figure 3:**
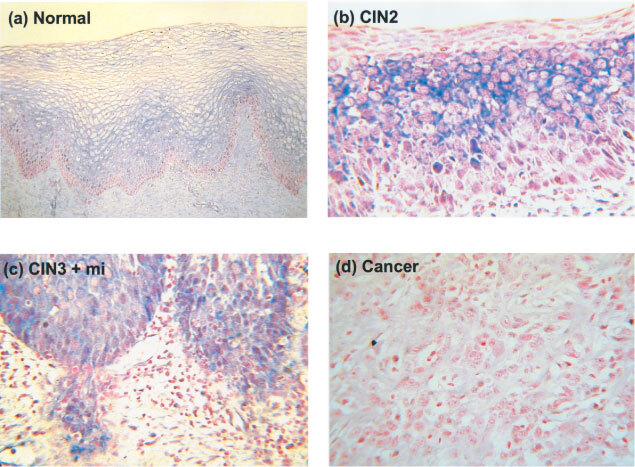
Immunohistochemical staining with FHIT antibody. (**A**) Normal epithelium (positive control) ×100, (**B**) a positive CIN2 lesion×350, (**C**) a positive CIN3 lesion showing microinvasion×350 and (**D**) a negative squamous cell carcinoma×350. Stromal cells present in all sections were used as an internal negative control.

shows the FHIT protein staining criteria used in grading individual lesions. FHIT protein expression was readily detectable in all normal epithelium and CIN1 lesions and in most CIN2/3 lesions but was totally absent in four CIN3 lesions (Table 2B). One of these was associated with HPV18 but the remaining three contained no detectable HR HPV. After adjusting for HR HPV, abnormal FHIT protein expression (reduced or absent) was also found to be associated with increasing CIN grade (*P*_trend_=0.004).

## DISCUSSION

A full length FHIT transcript was detected in all normal cervical tissue samples and in all but 3% (7 out of 223) of CIN lesions (Figure 1, Table 2A). The precise significance of its detection in almost all CIN lesions is unclear since it could have been derived from abnormal and/or adjacent normal epithelium. The presence of abnormal FHIT transcripts could be clearly identified and their nature defined by nucleic acid sequencing. They were found to be generated by alternative splice site usage and cryptic alternative splice sites were identified in exons 5, 7 and 10 and in introns 5 and 7 (Figure 2). Our results show that abnormal transcripts can be found in both histologically normal and abnormal tissues (Table 2A).

When compared against stromal cells within the same section, expression of FHIT protein in epithelial cells was found in all normal tissues and low grade CIN1 as well as in most high grade CIN2/3 lesions (Table 2B, Figure 3). This is to be expected if full length FHIT transcripts are expressed in most CIN lesions (Figure 1, Table 2A). However, abnormal FHIT protein expression was found in a proportion (17%) of exclusively CIN2 and CIN3 lesions and interestingly, three of the four CIN3 lesions which showed no FHIT protein expression also had no detectable HR HPV. Similar abnormal FHIT protein expression has previously been found in 52% of high grade lesions associated with cancer but only in 8% of those not associated with cancer (Connolly *et al*, 2000). This suggests that these CIN2/3 lesions could carry an increased risk of malignancy.

Recent evidence suggests that FHIT instability may play a synergistic role with HR HPV in the pathogenesis of high grade cervical lesions (Butler *et al*, 2000). HPV16 genomes have been found integrated in the vicinity of the FHIT coding sequence and the presence of HPV16 DNA was associated with LOH in the FHIT region (Muller *et al*, 1998; Butler *et al*, 2000; Yoshino *et al*, 2000). Detailed typing of HR HPV in our study did not show any associations between abnormal FHIT transcript expression and presence of specific HR HPV types or all HR HPV taken together. This is consistent with a negative correlation found between the presence of HPV E6/E7 transcripts and abnormal FHIT transcripts in cervical cancers (Segawa *et al*, 1999).

Our results indicate that, after adjusting for HR HPV, abnormal FHIT transcript expression (*P*_trend_<0.001) or abnormal FHIT protein expression (*P*_trend_<0.004) represents a risk factor for the development of high grade CIN lesions. This suggests that genomic instability at the FHIT locus induced by other risk factors (e.g. cigarette smoking, alcohol consumption or concurrent chronic inflammation) could account for the development of those high grade cervical lesions which are HR HPV negative and which may have a lower risk of cancer and tend to regress in the absence of persistent HR-HPV infection. For clinical or epidemiological purposes, testing for FHIT protein, rather than FHIT transcripts, would have obvious advantages since it can be carried out using low cost and simple procedures on routine paraffin sections.
